# Intracellular Delivery of Nanoparticles and DNAs by IR9 Cell-penetrating Peptides

**DOI:** 10.1371/journal.pone.0064205

**Published:** 2013-05-28

**Authors:** Betty R. Liu, Ji-Sing Liou, Yue-Wern Huang, Robert S. Aronstam, Han-Jung Lee

**Affiliations:** 1 Department of Natural Resources and Environmental Studies, National Dong Hwa University, Hualien, Taiwan; 2 Department of Biological Sciences, Missouri University of Science and Technology, Rolla, Missouri, United States of America; University of Pécs Medical School, Hungary

## Abstract

Cell-penetrating peptides (CPPs) comprised of basic amino residues are able to cross cytoplasmic membranes and are able to deliver biologically active molecules inside cells. However, CPP/cargo entrapment in endosome limits biomedical utility as cargoes are destroyed in the acidic environment. In this study, we demonstrate protein transduction of a novel CPP comprised of an INF7 fusion peptide and nona-arginine (designated IR9). IR9 noncovalently interacts with quantum dots (QDs) and DNAs to form stable IR9/QD and IR9/DNA complexes which are capable of entering human A549 cells. Zeta-potentials were a better predictor of transduction efficiency than gel shift analysis, emphasizing the importance of electrostatic interactions of CPP/cargo complexes with plasma membranes. Mechanistic studies revealed that IR9, IR9/QD and IR9/DNA complexes may enter cells by endocytosis. Further, IR9, IR9/QD and IR9/DNA complexes were not cytotoxic at concentrations below 30, 5 and 20.1 µM, respectively. Without labor intensive production of fusion proteins from prokaryotes, these results indicate that IR9 could be a safe carrier of genes and drugs in biomedical applications.

## Introduction

The cell membrane is a permeable barrier that protects living cells from the extracellular environment by controlling the movement of materials into and out of cells. The cytoplasmic membrane mediates a wide range of essential processes, including environmental sensing, nutrient uptake, cellular morphogenesis, secretion and cell wall biogenesis [1]. The importance of the plasma membrane is reflected by the fact that most pharmaceutical drugs target plasma membrane components [2]. Transport of exogenous molecules across this barrier is complex and is influenced by phospholipid, glycolipid, cholesterol and protein composition. Membrane permeability depends on specific membrane transporters as well as the size and polarity of molecules of interest. In the absence of specific transporters, the membrane only allows the movement of small hydrophobic molecules into the cell [3]. Large hydrophilic drugs and biological macromolecules, including DNAs, RNAs and proteins, do not cross cell membranes freely.

Cell-penetrating peptides (CPPs, also known as protein transduction domains) are a group of short peptides capable of traversing cell membrane and delivering a variety of cargoes into living cells [3]–[8]. They were originally derived from the viral transactivation of transcription (Tat) protein that is capable of crossing cell membranes [9], [10]. A basic amino acid-rich region of the truncated Tat protein was identified as the domain responsible for penetrating cell membranes and accumulating in cell nuclei [11]. During the last 15 years, more than 100 varieties of CPPs have been reported [12], and 843 CPPs are catalogued on the CPPsite (http://crdd.osdd.net/raghava/cppsite/) [13]. The essential feature of CPPs is the ability to transport other molecules into cells. CPPs include amphipathic, hydrophobic and cationic peptides [14]. CPPs can be classified into three major families: protein-derived, synthetic and chimeric [12]. For instance, Tat and penetratin, two of the first CPPs discovered, are protein-derived [4]. Nona-arginine (R9) and the model amphipathic peptide do not have any natural parent proteins and belong to the synthetic family. Members of the chimeric family incorporate various functional domains of natural proteins, such as Pep-1 and transportan [12]. Each family can be divided into several subgroups based on their origin or sequence characteristics.

In recent years, CPPs have been exploited to deliver biologically active molecules into cells and are one of the most promising tools in therapeutics [15]. Recently, more than 20 clinical trials are using CPPs to deliver macromolecular drug conjugates into patients with various diseases [6]. CPPs are capable of carrying a wide spectrum of cargo molecules, including many types of proteins, nucleic acids, peptide nucleic acids, cytotoxic drugs, inorganic particles and liposomes [4], [6], [16]. CPPs can deliver cargoes with sizes up to 200 nm in diameter [17]. Our laboratory has used arginine-rich CPPs to deliver proteins [18]–[26], DNAs [27]–[33], RNAs [34] and nanoparticles [35]–[39] into cells from various species. The internalization kinetics of CPPs is rapid, with a first-order rate constant of 0.007 sec^−1^ [40]. CPPs are not toxic to most cells [30]–[32], [35], [37]–[41], and the safety of CPPs has been demonstrated by a metabolic analysis [42]. Recently, a detailed study further confirmed that CPPs are nontoxic *in vitro* and nonimmunogenic *in vivo* [43].

Quantum dots (QDs) are inorganic semiconductor nanocrystals first introduced in the 1980s [44]. QDs have a size-range of 1 to 100 nm and consist of a few hundred to a few thousand atoms [45]. QDs are attractive alternatives to fluorescent proteins due to their colloidal nature, wide excitation properties, and narrow, size-dependent and composition-tunable emission spectral ranges. QD advantages over traditional fluorescent proteins include photostability, high fluorescence quantum yields, resistance to photobleaching and chemical degradation, and high levels of brightness [45]–[47]. Accordingly, QDs are increasingly being used in biomedical imaging studies, as cellular labels, intracellular sensors, deep-tissue and tumor targeting and imaging agents, and sensitizers for photodynamic therapy [48]. However, QDs do not readily enter cells, and aggregation often occurs before and after internalization [47], [49]. To overcome these limitations, QDs have been surface-modified by either covalent [50]–[52] or noncovalent [35]–[39] linkages with CPPs. Though CPP-facilitated delivery of QDs reduces the nonspecific absorption and side effects [48], QDs are still susceptible to entrapment and sequestration by endosomes or lysosomes in cells.

Transduction enhancers and endosomolytic agents have been employed to improve CPP transduction efficiency and to overcome endosomal/lysosomal entrapment [38], [53]–[55]. Most enhancers, such as pyrenebutyrate [53] and dimethyl sulfoxide (DMSO) [38], [54], either increase the net hydrophobicity of CPPs or increase membrane permeability, while chloroquine is a lysosomotropic agent that prevents lysosomal trapping [55]. Insofar as endocytosis is one of the primary mechanisms of cellular uptake of CPPs, quick release from endocytic vesicles into the cytosol is essential to preserve biological activity of the cargoes [3], [6]–[8]. Several lysosomotropic peptides, also called endosome-disruptive peptides or membrane destabilizing peptides, have been derived from viral and bacterial toxins [7]. These peptides trigger endosomal acidification that leads to cargo escape into the cytosol [56]–[58]. INF7 peptide, a glutamic acid-enriched influenza virus hemagglutinin-2 (HA2) analogue, has been shown to be a particularly potent fusion peptide [59], as was used in the present study.

The aims of this study were to (1) create a chimeric IR9 CPP containing both INF7 fusion peptide and R9 (designated IR9), (2) evaluate the transduction of IR9 for cellular delivery of QDs and plasmid DNAs and (3) determine the mechanism of CPP-mediated uptake of QDs and DNAs in cells. To achieve these goals, we synthesized IR9 and examined the intracellular delivery of IR9, IR9/QD and IR9/DNA using live cell imaging and flow cytometry. To understand the relationship between transduction efficiency and IR9/cargo ratios, the charging state and electrostatic interactions of IR9/cargo complexes were characterized using a zeta-potential analyzer. To elucidate the uptake mechanisms of IR9 and IR9/cargo complexes, physical and pharmacological inhibitors were used to block specific endocytic pathways. Finally, the cytotoxicity of IR9 and IR9/cargo complexes was assessed.

## Materials and Methods

### Cell Culture

Human bronchoalveolar carcinoma A549 cells (American Type Culture Collection, Manassas, VA, USA; CCL-185) were maintained in Roswell Park Memorial Institute (RPMI) 1640 medium (Gibco, Invitrogen, Carlsbad, CA, USA) supplemented with 10% (v/v) bovine serum (Gibco) [35]. Living cells were determined by propidium iodide stain. Cells were washed with phosphate buffered saline (PBS) three times before and after each treatment. The culture medium was switched to RPMI 1640 medium supplemented with 1% serum during incubation with IR9-FITC, IR9/QD or IR9/DNA.

### QDs and Preparation of Peptides

Carboxyl-functionalized CdSe/ZnS QDs eFluor 525NC (green fluorescent QD) and eFluor 625NC (red fluorescent QD; denoted as QDr) possess the maximal emission peak wavelengths of 525 and 625 nm, respectively (eBioscience, San Diego, CA, USA). IR9 peptide (GLFEAIEGFIENGWEGMIDGWYGRRRRRRRRR) of 92.9% purity was chemically synthesized (Genomics, Taipei, Taiwan). IR9-FITC peptide of 90.2% purity contained the fluorescein isothiocyanate (FITC) at the N-terminus (Genomics). The molecular masses of IR9 and IR9-FITC are 3996.6 and 4499.1 Dalton, respectively.

### Gel Retardation Assay

To prepare IR9/QD complexes, IR9 peptide was mixed with 2 µM of QDs at molecular ratios of 0 (QD only), 15, 30, 60, 90 and 120 in PBS, and incubated at 37°C for 2 h. IR9/QD complexes were analyzed by electrophoresis on a 0.5% agarose gel (Multi ABgarose, Thermo Fisher Scientific, Waltham, MA, USA) in 0.5 × TAE (40 mM of Tris-acetate and 1 mM of EDTA, pH 8.0) buffer at 100 V for 40 min, as previously described [38]. To prepare IR9/DNA complexes, different amounts of IR9 were mixed with 3 µg of the pEGFP-N1 plasmid (Clontech, Mountain View, CA, USA) encoding the enhanced green fluorescent protein (*EGFP*) reporter gene at various molar nitrogen/phosphate (NH_3_^+^/PO_4_^−^ or N/P) ratios of 0 (DNA only), 0.5, 1, 1.5, 2, 2.5, 3, 6, 9, 12 and 15. After 2 h incubation, the IR9/DNA mixtures were analyzed by electrophoresis on a 0.5% agarose gel at 100 V for 40 min and stained with SYBR® Green 1 (Molecular Probes, Eugene, OR, USA), as previously described [31]. Images were captured using a Typhoon Trio imager (GE Healthcare, Piscataway, NJ, USA) with the excitation wavelength of 532 nm (SYAG laser) and with the emission of 532 nm. Data were analyzed using ImageQuant TL 7.0 software (GE Healthcare).

### Noncovalent QD Transduction

In the protein transduction experiments, different amounts of IR9-FITC peptide (0, 1, 5, 10, 30 and 60 µM) were incubated with human A549 cells for 1 h, and cells were then analyzed with flow cytometry. Cells treated with PBS, FITC (Sigma-Aldrich, St. Louis, MO, USA) or a non-CPP (casein-FITC, Sigma-Aldrich) served as negative controls. In the kinetic study of transduction, 5 µM of IR9-FITC were added to cells for 0, 1, 5, 10, 30 and 60 min at 37°C. To determine subcellular colocalization of IR9-FITC, organelle-specific fluorescent trackers Hoechst 33342 (Invitrogen) and LysoTracker DND-99 (Invitrogen) were utilized to visualize nuclei and lysosomes, respectively, according to the manufacturer’s instructions. Cells were treated with 5 µM of IR9-FITC for 1 h and stained with both trackers [38].

For the transduction of noncovalent IR9/cargo complexes, 1, 5, 10, 30 and 60 µM of IR9 peptide were mixed with QDs at a molecular ratio of 60 at 37°C for 2 h. IR9/QD complexes were then incubated with the cells at 37°C for 1 h. To study transduction kinetics, cells were treated with IR9/QD complexes prepared at a molecular ratio of 60 for 0, 1, 5, 10, 30, 60 min, 12 h and 24 h at 37°C. In other experiments with high molecular ratios, cells were treated with IR9/QD complexes prepared at molecular ratios of 120 and 240 for 24 h followed by analysis with a confocal microscope or a flow cytometer. To test QD dissociation from CPPs, 5 µM of IR9-FITC was mixed with 2.6 nM of QDr at 37°C for 2 h, and then incubated with cells for 24 h. Following incubation, IR9-FITC/QDr complexes were removed and cells were stained with Hoechst 33342, followed by observation using a confocal microscope. Lysosomal escape was conducted by adding 25 µM of chloroquine (Sigma-Aldrich) to cells previously treated with IR9/QD complexes for 24 h. The cells were then stained with LysoTracker DND-99 and Hoechst 33342.

To evaluate the role of endocytosis in complex transduction, physical and pharmacological endocytic modulators were used [38]. Cells were incubated at 4°C for 30 min to deplete energy required by all endocytic pathways; IR9-FITC, IR9/QD or IR9/DNA complexes were then incubated with the cells at 4°C. To analyze the role of macropinocytosis in complex transduction, cells were treated with 10 µM of cytochalasin D (CytD), 100 µM of 5-(*N*-ethyl-*N*-isopropyl)-amiloride (EIPA), 5 µg/ml of filipin or 10 µM of nocodazole (Sigma-Aldrich) for 1 h to block F-actin rearrangements, macropinocytosis, caveolae-dependent endocytosis or clathrin-dependent endocytosis, respectively. The cells were then treated with IR9-FITC, IR9/QD or IR9/DNA complexes and uptake determined.

### DNA Delivery Mediated by IR9 and Functional Gene Assay

To prepare fluorescence-labeled DNAs, the pBlueScript-SK+ plasmid (Agilent Technologies, Santa Clara, CA, USA) was labeled with the *Label*IT Cyanine 3 (Cy3) nucleic acid labeling kit (Mirus Bio, Madison, WI, USA) [27]. Cells were seeded at a density of 1 × 10^4^ per well of 96-well plates. Three µg of Cy3-labeled pBlueScript-SK+ plasmid DNA was incubated with IR9 at N/P ratios of 0 (Cy3-labeled DNA only), 1, 3, 6 and 9 in a final volume of 100 µL for 2 h at 37°C. The IR9/Cy3-labeled DNA complexes were added to cells in the 96-well plates, and the plates were incubated for 1 h at 37°C. The cells were washed three times with PBS to remove free IR9/Cy3-labeled DNA complexes, and then staining with Hoechst 33342.

In a functional gene assay, cells were treated with either 3 µg of the pEGFP-N1 plasmid DNA mixed with IR9 at N/P ratios of 0 (control), 3, 6, 9 or 12. These complexes were transferred to cells in each well for 1 h at 37°C. The cells were then washed three times with PBS. The cells were supplemented with 100 µL of 10% serum-containing medium and incubated at 37°C for 48 h, and then stained with Hoechst 33342 and observed using a confocal microscope [30], [31].

### Fluorescent and Confocal Microscopy

Fluorescent and bright-field images were recorded using a BD Pathway 435 bioimaging system (BD Biosciences, Franklin Lakes, NJ, USA) which includes both the fluorescent and confocal microscopic sets without a pinhole [38]. Excitation filters were at 377/50, 482/35 and 543/22 nm for blue, green and red fluorescence, respectively. Emission filters were at 435LP (long-pass), 536/40 and 593/40 nm for blue (BFP), green (GFP) and red fluorescent protein (RFP) channels, respectively. Confocal images were also obtained using the TCS SP5 II confocal microscope system (Leica, Wetzlar, Germany). The parameters for this confocal microscopy were as follows: excitation at 405 nm and emission at 435–480 nm for the detection of BFP; excitation at 488 nm and emission at 495–540 nm for the detection of GFP; and excitation at 543 nm and emission at 590–665 nm for the detection of RFP. Relative intensities of fluorescent images were quantified using UN-SCAN-IT software (Silk Scientific, Orem, UT, USA). Bright-field microscopy was used to assess cell morphology.

### Flow Cytometric Analysis

Cells were seeded at a density of 2.5 × 10^5^ per well of 24-well plates. Cells in the control and experimental groups treated with IR9-FITC or IR9/cargo complexes were harvested and counted using a Cytomics FC500 flow cytometer (Beckman Coulter, Fullerton, CA, USA) with a FL1 filter for GFP detection [19], [24]. Data were analyzed using CXP software (Beckman Coulter).

### Zeta-potential Analysis

IR9 (12.4 µM), QD (100 nM), pEGFP-N1 plasmid DNA (7 µg), IR9/QD complexes formed at molecular ratios of 60, 120 and 240, and IR9/DNA complexes formed at N/P ratios of 9 and 12 were prepared in double deionized water. Each solution was temperature-equilibrated at 25°C for 120 sec in a zeta cell. Zeta-potentials of samples were measured using a Zetasizer Nano ZS (Malvern Instruments, Worcestershire, UK) and analyzed using Zetasizer software 6.30 (Malvern) [39]. The correlation coefficient analysis between zeta-potential and protein transduction efficiency was plotted using SigmaPlot software (Systat, Chicago, IL, USA).

### Cytotoxicity Measurement

Cells were treated with 1, 5, 10, 30 and 60 µM of IR9-FITC, IR9, IR9/QD or IR9-FITC/QDr complexes prepared at CPP/probe ratios of 60∶1, 120∶1 and 240∶1 for 24 h at 37°C. To measure the transduction of IR9/DNA complexes, cells were treated with IR9/pEGFP-N1 complexes prepared at N/P ratios of 0 (DNA only), 1, 3, 6, 9 and 12 for 24 h at 37°C. Cells without any treatment served as a negative control, while cells treated with 100% DMSO served as a positive control [60]. Cell viability was determined using the 1-(4,5-dimethylthiazol-2-yl)-3,5-diphenylformazan (MTT) assay [31].

### Statistical Analysis

Results are expressed as mean ± standard deviation (SD). Mean values and SDs were calculated from at least three independent experiments of triplicates per treatment group. Comparisons between the control and treated groups were performed by the Student’s *t*-test using levels of statistical significance of *P*<0.05 (*, α, β) and 0.01 (**, αα, ββ), as indicated.

## Results

**Figure 1:**
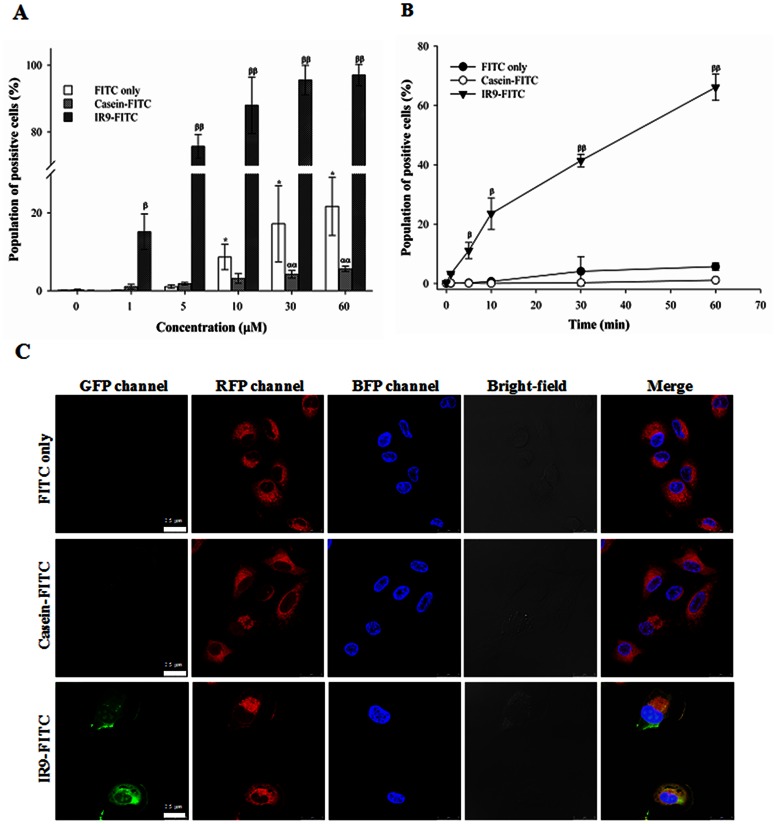
Internalization of IR9 into human A549 cells. (A) Flow cytometric analysis of concentration-dependent entry of IR9 into human A549 cells. Cells were treated with FITC, casein-FITC, or 0, 1, 5, 10, 30 and 60 µM of IR9-FITC for 1 h, and the fluorescent intensity was analyzed using a Cytomics FC500 flow cytometer (Beckman Coulter). Data are presented as mean ± SD from 5 independent experiments in each treatment group. (B) Flow cytometric analysis of time course-dependent entry of IR9 into cells. Cells were treated with FITC, casein-FITC, or 5 µM of IR9-FITC for 0, 1, 5, 10, 30 and 60 min. The fluorescent intensity was analyzed using a flow cytometer. Significant differences of *P*<0.05 (*, α, β) and *P*<0.01 (**, αα, ββ) are indicated. Data are presented as mean ± SD from 3 independent experiments in each treatment group. (C) Subcellular colocalization of IR9 after entry into cells. Cells were treated with either FITC and casein-FITC as controls or 5 µM of IR9-FITC as the experimental group for 1 h, and then stained with Hoechst 33342 and LysoTracker DND-99. Images were taken using a Leica confocal microscope system. Cell morphologies are shown as bright-field images. GFP, RFP and BFP channels revealed the distribution of FITC-labeled objects, lysosomes and nuclei, respectively. The merged images combined BFP, RFP and GFP channels at a magnification of 1,260×. Overlaps between peptides and organelle trackers exhibit yellow color in merged GFP and RFP images, while those between objects and nuclei show cyan color in merged GFP and BFP images. Scale bar is 25 µm.

To determine the kinetics of protein transduction of the novel IR9 peptide, human A549 cells were treated with FITC, casein-FITC, or various amounts of IR9-FITC peptide and analyzed by flow cytometry. Cellular internalization of IR9-FITC peptide was concentration-dependent (Fig. 1A). The kinetic experiment showed that internalization of IR9-FITC is time-dependent, entering into cells within 1 min and reaching a stationary phase at 60 min (Fig. 1B). The IR9-FITC was distributed evenly throughout the cytosol. Merged images indicated that IR9-FITC is colocalized with lysosomes (Fig. 1C). These results demonstrate that IR9 possesses protein transduction ability.

**Figure 2:**
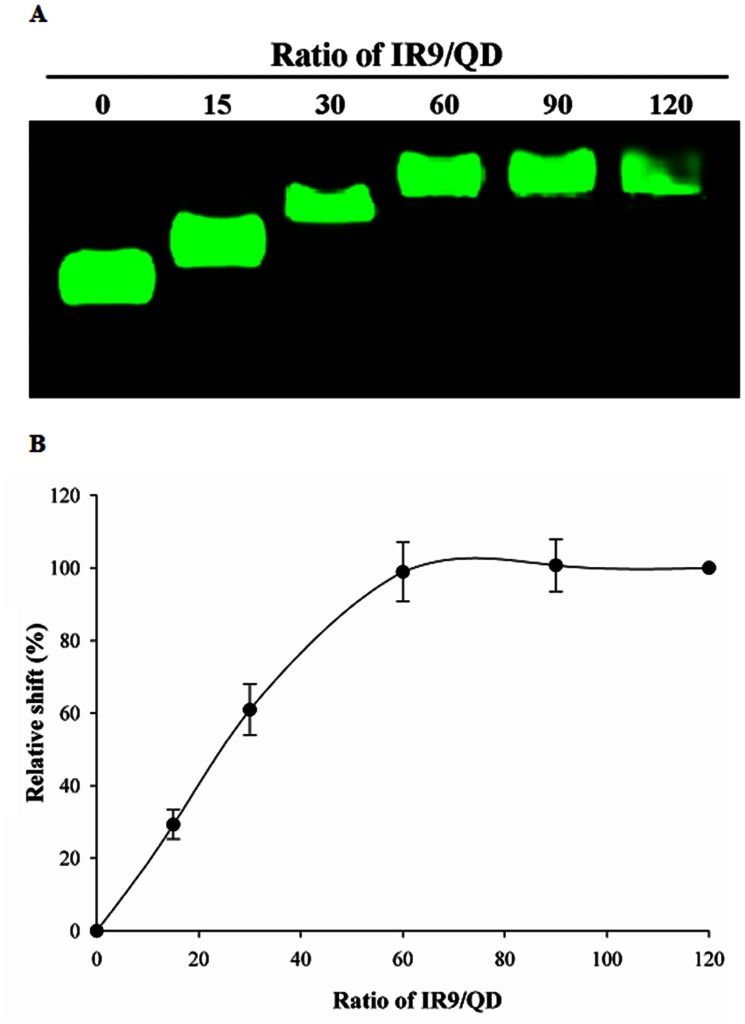
Noncovalent interactions between IR9 and QDs *in vitro*. (A) Gel retardation assay revealing stable interactions between IR9 and QDs. Different amounts of IR9 were mixed with QDs at molecular ratios of 0 (QD only), 15, 30, 60, 90 and 120. IR9/QD mixtures were subjected to electrophoresis on a 0.5% agarose gel. Fluorescence of QDs was visualized at 532 nm using a Typhoon Trio imager (GE Healthcare). (B) Relative shift of IR9/QD complexes formed at different IR9/QD ratios. Data are presented as mean ± SD from 6 independent experiments in each treatment group.

Noncovalent interactions between IR9 and QDs were confirmed in an agarose-based gel retardation assay (Fig. 2A). Semi-quantitative analysis revealed ratio-dependent interactions of IR9/QD complexes reaching a plateau at a molecular ratio of 60 (Fig. 2B). Accordingly, this ratio was used in subsequent experiments.

**Figure 3:**
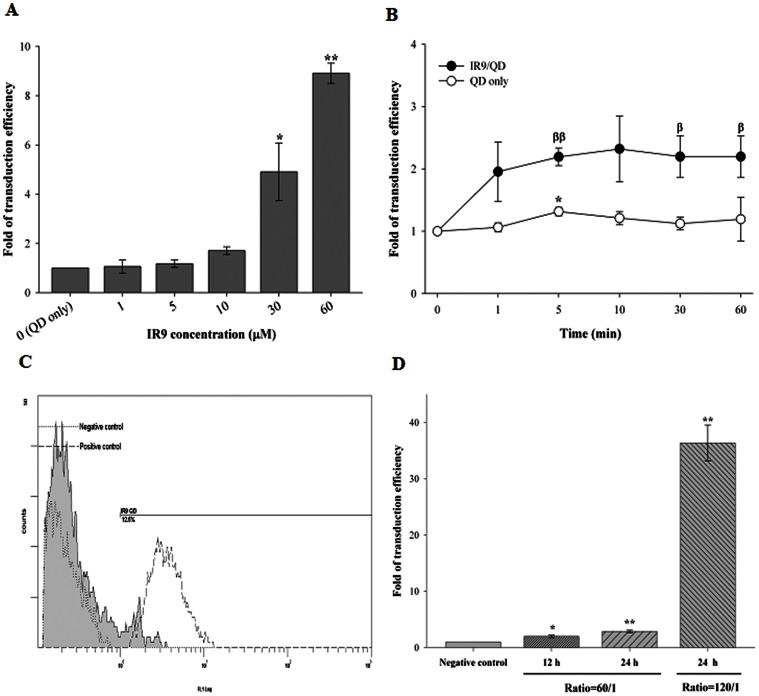
Flow cytometric analysis of cellular internalization of IR9/QD complexes. (A) Concentration-dependent internalization of IR9/QD complexes into cells. Different amounts of IR9 (1, 5, 10, 30 and 60 µM) were mixed with QDs at a molecular ratio of 60. IR9/QD complexes were transferred onto cells and incubated for 1 h. The fluorescent intensity was analyzed using a flow cytometer. Cells treated with QDs alone served as a negative control. Data are presented as mean ± SD from 3 independent experiments in each treatment group. (B) Time-dependent internalization of IR9/QD complexes into cells. Five µM IR9 were mixed with QDs at a molecular ratio of 60 and then incubated with cells for 0 (negative control), 1, 5, 10, 30 and 60 min, as indicated. Data are presented as mean ± SD from 3 independent experiments in each treatment group. (C) Merged flow cytometric plots of cellular internalization of IR9/QD complexes. Cells were treated with IR9/QD complexes prepared at a molecular ratio of 120 at 37°C for 24 h (gray area). Results from the negative control are shown by the dotted line at the left, while results from the positive controls (5 µM of IR9-FITC) are shown by dashed line. (D) Cellular internalization comparison of IR9/QD complexes at different combined ratios. Cells were treated with different molecular ratios (60 and 120) of IR9/QD complexes for the indicated periods of time (12 and 24 h). Significant differences of *P*<0.05 (*, β) and *P*<0.01 (**, ββ) are indicated. Data are presented as mean ± SD from 3 independent experiments in each treatment group.

To study IR9-dependent cellular internalization, cells were treated with IR9/QD complexes containing different amounts of IR9 but a fixed ratio of 60 with QDs. The transduction of IR9 was concentration-dependent (Fig. 3A), and intracellular accumulation of IR9/QD complexes could be detected within 1 min (Fig. 3B). We noted stronger fluorescence at a molecular ratio of 60 of IR9/QD complexes in 24 h compared to that in 12 h (Fig. 3C and D). A much larger fraction of the cells internalized IR9/QD complexes at a molecular ratio of 120 compared to that at a ratio of 60 (Fig. 3C and D). These data suggest that IR9 not only can interact with QDs to form stable IR9/QD complexes (Fig. 2), but also can deliver QDs into cells (Fig. 3).

**Figure 4:**
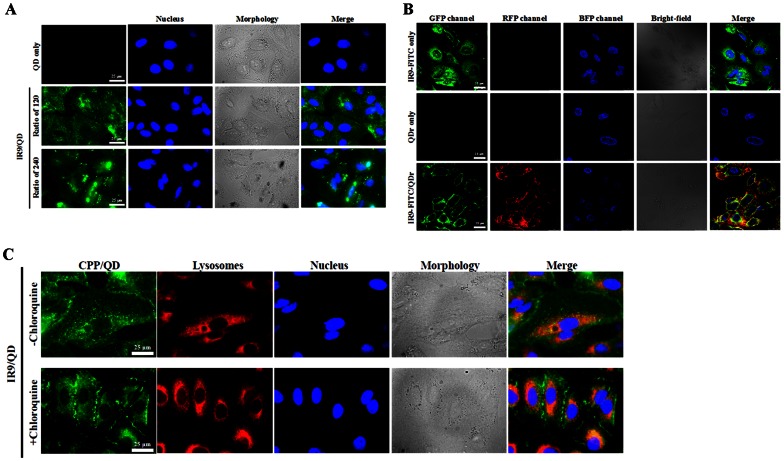
Confocal microscopy of intracellular delivery of IR/QD complexes into A549 cells. (A) Images of A549 cells treated with IR9/QD complexes prepared at various combination ratios. IR9 was mixed with QDs at molecular ratios of 120 and 240, and then incubated with cells for 24 h at 37°C. The cells were stained with Hoechst 33342 and then observed using a BD Pathway 435 System (BD Biosciences) at a magnification of 600×. GFP and BFP channels revealed the distribution of QDs and nuclei, respectively. Cell morphologies are shown in bright-field images. Overlaps between QDs and nuclei are cyan in merged GFP and BFP images. Scale bar is 25 µm. (B) Association between IR9 and QDs after cellular internalization. Cells were treated with IR9-FITC and QDr as controls. Five µM of IR9-FITC was mixed with 2.6 nM of QDr at 37°C for 2 h, and IR9-FITC/QDr complexes were added to cells for 24 h at 37°C. Cells were stained with Hoechst 33342 and observed using a Leica confocal microscope system at a magnification of 1,260×. GFP, RFP and BFP channels revealed the distribution of IR9-FITC, QDr and nuclei, respectively. Overlaps between peptides and QDr were yellow in merged GFP and RFP images. (C) Subcellular colocalization of IR9-delivered QDs. Cells were treated with IR9/QD complexes prepared at a molecular ratio of 120 for 24 h in the absence or presence of 25 µM chloroquine. Cells were stained with LysoTracker DND-99 and Hoechst 33342, and images were then observed using a BD Pathway System at a magnification of 600×. GFP, RFP and BFP channels displayed the distribution of QDs, lysosomes and nuclei, respectively. Overlaps between QDs and lysosomes were yellow/orange color in merged GFP and RFP images.

To reveal the subcellular localization of IR9-delivered QDs, cells were treated with QDs only or IR9/QD complexes at various combination ratios, and then stained with a nucleus-specific fluorescent marker Hoechst 33342. The IR9-delivered QDs were evenly distributed in the cytosol (Fig. 4A). To address whether CPPs and cargos dissociate after cellular internalization, cells were treated with IR9-FITC/QDr complexes, and then stained with Hoechst 33342. The merged image showed that QDs were largely colocalized with IR9 within cells (Fig. 4B). This indicates that QDs remain associated with IR9 following cellular entry. Additionally, lysosomal escape was affected using the lysosomotropic agent chloroquine. The cells were then stained with nucleus- and lysosome-specific fluorescent markers (Hoechst 33342 and LysoTracker DND-99, respectively). The QDs initially colocalized with lysosomes, and chloroquine facilitated cytoplasmic distribution of IR9-delivered QDs (Fig. 4C). Green puncta were observed in the cells treated with IR9/QD complexes, but no green puncta were seen in the cells treated with IR9/QD complexes and chloroquine (Fig. 4C). These results suggest that intracellular delivery of IR9/QD complexes may involve an endocytic pathway, and chloroquine promotes lysosomal escape.

**Figure 5:**
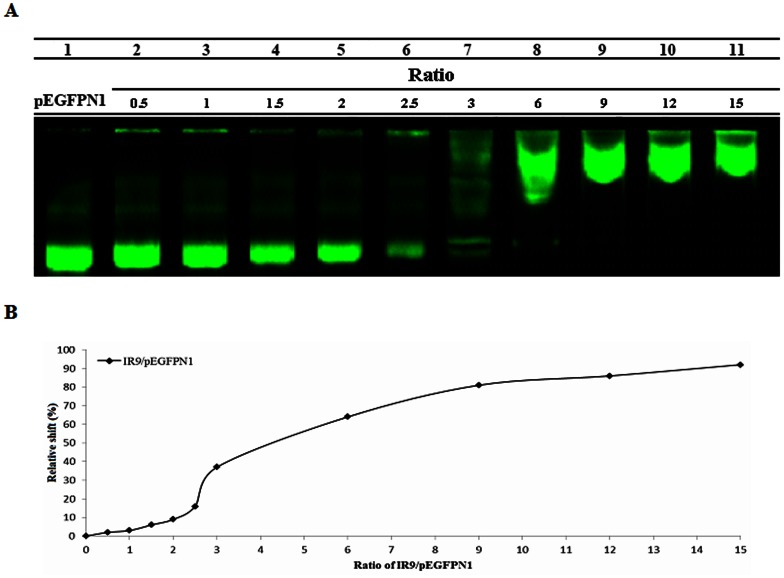
Noncovalent interactions between IR9 and plasmid DNAs *in vitro*. (A) Gel retardation assay of IR9/DNA complexes. Different amounts of IR9 were mixed with the pPEGFP-N1 plasmid at molecular ratios of 0 (DNA only), 0.5, 1, 1.5, 2, 2.5, 3, 6, 9, 12 and 15, as indicated. After a 2 h incubation, IR9/DNA complexes were analyzed by electrophoresis on a 0.5% agarose gel and stained by SYBR® Green 1. (B) The relative shift percentage (*y*-axis) as a function of IR9/DNA ratio.

To assess whether IR9 can deliver genes into cells, we switched QD cargoes to plasmid DNAs. A gel retardation assay was conducted with IR9 and the pEGFP-N1 plasmid DNA. Results indicated that IR9 noncovalently associates with DNAs to form stable IR9/DNA complexes (Fig. 5A). DNA mobility decreased as the amount of IR9 increased (Fig. 5B). Thus, IR9 interacts with DNAs to form stable IR9/DNA complexes *in vitro*.

**Figure 6:**
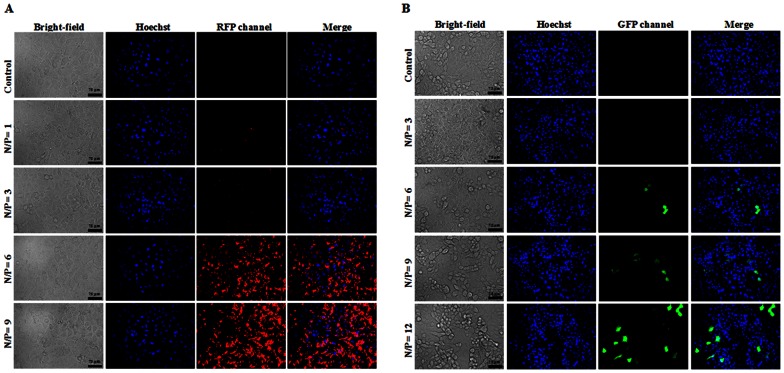
Fluorescent microscopy of delivery of IR/DNA complexes into A549 cells. (A) Images of IR9-mediated delivery of the Cy3-labeled plasmid DNAs into cells. A549 cells were treated with IR9/Cy3-labeled pBlueScript-SK+ complexes prepared at N/P ratios of 0 (Cy3-labeled DNAs), 1, 3, 6 and 9 and incubated for 1 h at 37°C. The cells were stained with Hoechst 33342 and then observed using a BD Pathway System at a magnification of 200×. Cell morphologies are shown in bright-field images. The merged images combined BFP and RFP channels reveal the distribution of nuclei and Cy3-labeled DNAs, respectively. Overlaps between nuclei and Cy3-labeled DNAs are purple in merged BFP and RFP images. Scale bar is 75 µm. (B) IR9-mediated delivery of EGFP reporter gene into cells. Cells were treated with IR9/pEGFP-N1 complexes prepared at N/P ratios of 0 (DNA only), 3, 6, 9 and 12 and incubated for 1 h at 37°C. The cells were washed with PBS several times to remove free IR9/DNA complexes, and then supplemented with 200 µL of 10% serum-containing medium for 48 h at 37°C. The cells were stained with Hoechst 33342 and observed using a BD Pathway System at a magnification of 200×. The merged images from BFP and GFP channels indicate the distribution of nuclei and *EGFP* reporter gene-expressing cells, respectively. Overlaps between nuclei and reporter gene-expressing cells are cyan in merged BFP and GFP images.

To determine whether DNA plasmids are delivered by IR9 into cells, A549 cells were treated with IR9/Cy3-labeled pBlueScript-SK+ plasmid DNA complexes at various N/P ratios, stained with Hoechst 33342, and then observed using a fluorescent microscope. No signal was detected in cells treated with Cy3-labeled DNA alone (Fig. 6A). In contrast, red fluorescence was observed in cells treated with IR9/Cy3-labeled DNA complexes when N/P was >1. This indicates that IR9 can transport DNA into cells. In the functional gene assay, cells were treated with IR9/pEGFP-N1 complexes at different N/P ratios and stained with Hoechst 33342. No signal was detected in cells treated with the pEGFP-N1 plasmid DNA encoding an *EGFP* reporter gene (Fig. 6B). In contrast, green fluorescence was observed in cells treated with IR9/DNA complexes when N/P was >6, indicating that plasmid DNAs delivered by IR9 can be actively expressed by cells.

**Figure 7:**
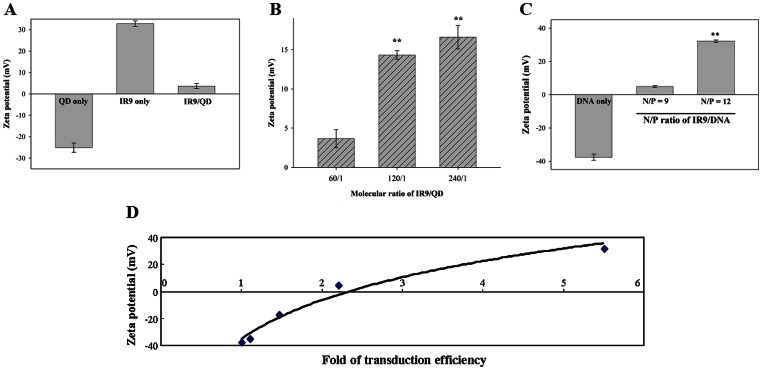
Zeta-potential measurements of QDs, IR9, DNAs and IR9/cargo complexes. (A) Zeta-potentials of QDs, IR9 and IR9/QD complexes. QDs, IR9 peptides or IR9/QD complexes prepared at a molecular ratio of 60 were liquefied with double deionized water and measured using a Zetasizer (Malvern Instruments). (B) Comparison of zeta-potentials of IR9/QD complexes prepared at molecular ratios of 60, 120 and 240, respectively. Samples were liquefied with double deionized water and measured using a zeta-potential analyzer. The value measured in IR9/QD complexes prepared at a molecular ratio of 60 served as the control. Zeta-potentials of IR9/QD complexes prepared at molecular ratios of 120 and 240 were compared to that of the control. Significant differences of *P*<0.01 (**) are indicated. Data are presented as mean ± SD from 3 independent experiments in each group. (C) Zeta-potentials of DNAs and IR9/DNA complexes. IR9 was mixed with the pEGFP-N1 plasmid at N/P ratios of 9 and 12, respectively. After forming complexes, DNA and IR9/DNA complexes were liquefied with double deionized water and measured using a zeta-potential analyzer. The value measured in IR9/DNA complexes at an N/P ratio of 9 served as the control. Significant differences of *P*<0.01 (**) are indicated. (D) The correlation coefficient analysis between zeta-potential and protein transduction efficiency. Cells were treated with IR9/DNA complexes prepared at N/P ratios of 0 (DNA only), 3, 6, 9 and 12 and then analyzed using a Zetasizer. Transduction efficiency was analyzed by the UN-SCAN-IT software. A logarithmic curve was plotted with zeta-potential (*y*-axis) against transduction efficiency (*x*-axis) using SigmaPlot software.

To understand the contribution of N/P ratio in transduction efficiencies of IR9/cargo complexes (i.e., Fig. 2 *versus* Fig. 3D and 4; and Fig. 5 *versus* Fig. 6), the charge state of IR9, cargo and IR9/cargo complexes were characterized using a zeta-potential analyzer. Zeta values of carboxyl-functionalized QDs and arginine-rich IR9 were −25.1±2.2 mV and 32.8±1.3 mV, respectively (Fig. 7A). The surface charge of IR9/QD complexes formed at a molecular ratio of 60 was near neutral (3.7±1.2 mV). However, zeta values of IR9/QD complexes were dramatically elevated at molecular ratios above 60 (Fig. 7B). This relationship was also observed with IR9/DNA complexes. While plasmid DNA has a zeta-potential of −37.6±1.9 mV, the zeta value of IR9/DNA complexes was 4.92±0.5 mV at an N/P ratio of 9 and 32.2±0.7 mV at an N/P ratio of 12 (Fig. 7C). A logarithmic curve was plotted with zeta value against protein transduction efficiency to generate an equation of *y*  = 41.676Ln(*x*) −35.16 with an R-squared value of 0.9737 (Fig. 7D). The correlation coefficient analysis demonstrated a high correlation between the zeta-potential and transduction efficiency of CPP/DNA complexes. Thus, in addition to gel shift ability, the electrostatic interactions of CPP/cargo complexes can be a predictor of transduction efficiency within the charge range tested.

**Figure 8:**
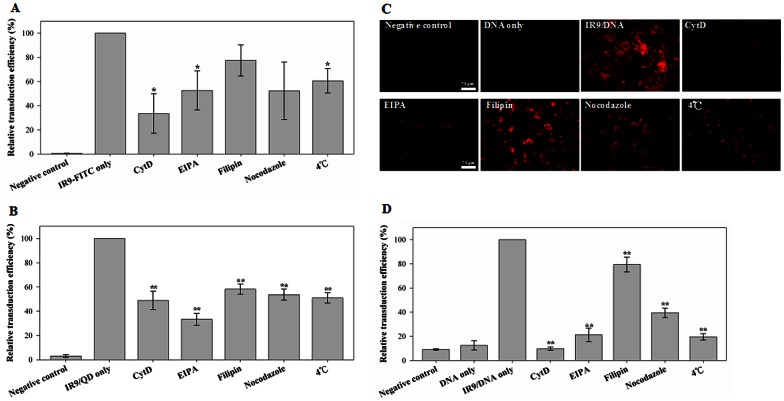
Mechanisms of cellular internalization of IR9 and IR9/cargo complexes. (A) Effect of inhibitors on cellular uptake of IR9-FITC. Cells were treated with PBS (negative control) or IR9-FITC in the absence or presence of the endocytic inhibitors CytD, EIPA, filipin, nocodazole and treatment of 4°C. After treatment for 1 h, the cells were analyzed by flow cytometry. (B) Effect of inhibitors on the cellular uptake of IR9/QD complexes. Cells were treated with PBS (negative control) or IR9/QD complexes in the absence or presence of endocytic inhibitors. (C) Cellular uptake of IR9/DNA complexes without or with inhibitors. Scale bar is 75 µm. (D) Effect of inhibitors on the cellular uptake of IR9/DNA complexes. Cells were treated with PBS (negative control), DNAs alone or IR9/DNA complexes in the absence or presence of endocytic inhibitors. Significant differences of *P*<0.05 (*) and *P*<0.01 (**) are indicated. Data are presented as mean ± SD from 3 independent experiments in each treatment group.

We used physical and pharmacological inhibitors to elucidate mechanisms of cellular internalization of IR9 and its associated cargoes. Cells were treated with PBS (as a negative control), IR9-FITC, IR9/QD or IR9/DNA complexes in the absence or presence of endocytic inhibitors, followed by flow cytometric analysis. We found that cellular uptake of IR9-FITC was sensitive to treatments of 4°C, CytD and EIPA (Fig. 8A). Cellular internalization of IR9/QD complexes was inhibited by treatments of 4°C, CytD, EIPA and nocodazole (Fig. 8B). Cellular entry of IR9/DNA complexes was sensitive to treatments of 4°C, CytD, EIPA, filipin and nocodazole (Fig. 8C and D). These results indicate that the classical energy-dependent endocytosis may be one of the main routes for cellular internalization of IR9 and IR9/cargo complexes.

**Figure 9:**
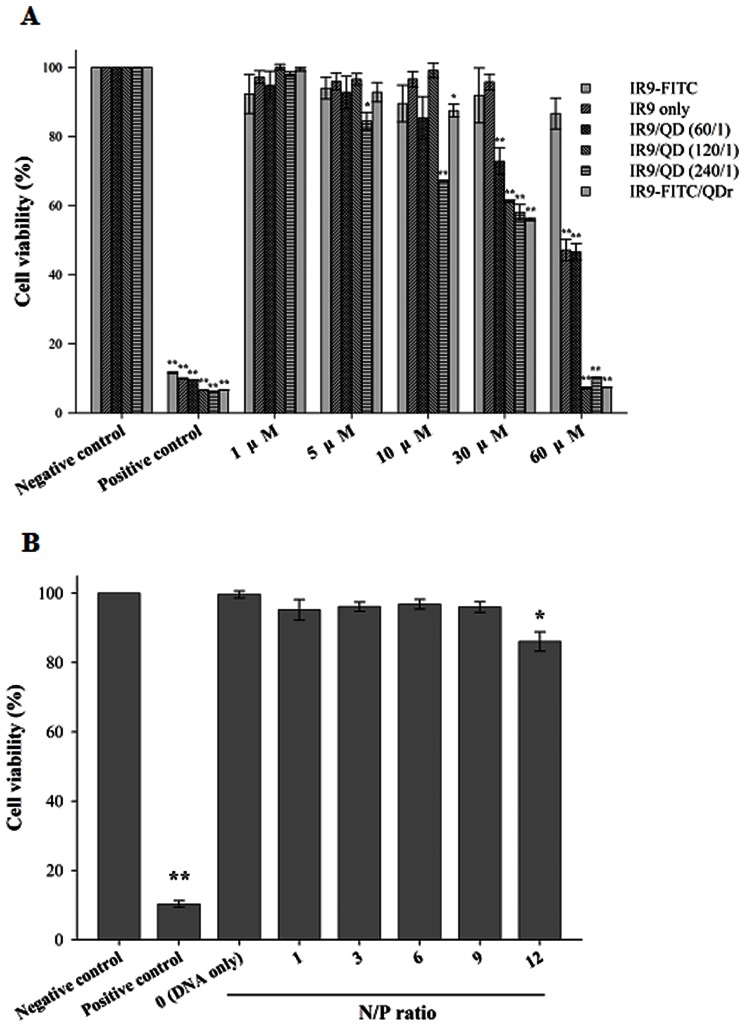
Cytotoxicity of IR9 and IR9/cargo complexes. (A) Influence of IR9-FITC, IR9 and IR9/QD complexes on A549 cell viability. Cells were treated with different concentrations (1, 5, 10, 30 and 60 µM) of IR9-FITC, IR9 or IR9/QD complexes prepared at molecular ratios of 60, 120 and 240, or IR9-FITC/QDr complexes. After a 24 h incubation at 37°C, mitochondrial succinate dehydrogenase activity was analyzed using the MTT assay. Cells treated with PBS and DMSO for 24 h served as negative and positive controls, respectively. (B) Influence of DNA and IR9/DNA complexes on cell viability. Cells were incubated with IR9/pEGFP-N1 complexes prepared at N/P ratios of 0 (DNA only), 1, 3, 6, 9 and 12 at 37°C for 24 h, as indicated. Cells treated with PBS and DMSO for 24 h served as negative and positive controls, respectively. The MTT assay was used to evaluate cell viability. Significant differences of *P*<0.05 (*) and *P*<0.01 (**) are indicated. Data are presented as mean ± SD from 3 independent experiments in each treatment group.

The MTT assay was performed to determine the effect of IR9-mediated cargo delivery on cell viability. Cells were treated with IR9-FITC, IR9, IR9/QD, IR9-FITC/QDr or IR9/DNA complexes for 24 h. IR9 alone and IR9/QD complexes were not cytotoxic at concentrations below 30 µM, while IR9/QD complexes above 5 µM at a molecular ratio of 240 were toxic (Fig. 9A). IR9/DNA complexes showed no cytotoxicity when N/P was <12, corresponding to concentrations below 20.1 µM (Fig. 9B).

## Discussion

In this study, we introduce and characterize a novel cell-penetrating peptide without labor intensive production of proteins from prokaryotes. IR9 is a chimeric molecule derived from fusion of synthetic nona-arginine and the fusogenic peptide INF7. IR9 can noncovalently interact with QDs and DNAs to form stable complexes and deliver both into human A549 cells. A high correlation is noted between zeta-potential and protein transduction efficiency of CPP/DNA complexes. Electrostatic interactions of IR9/cargo complexes with the plasma membrane play an important role in cellular internalization. Endocytosis may be one of the main routes for cellular uptake of IR9 and IR9/cargo complexes. Neither IR9 nor IR9/cargo complexes are cytotoxic at low concentrations. These properties indicate that IR9 may be a useful tool in the study of biological processes, including gene expression, as well as a delivery vector in biomedical applications.

Poor intracellular trafficking and endosomal release are major factors that reduce efficiency of CPP protein transduction mediated by endocytic pathways [3], [6], [8], [56]; endosomal entrapment can lead to enzymatic degradation of CPPs and their cargoes. Entrapment in endosomes or macropinosomes can be overcome by incorporating HA2 and INF7 peptides into CPPs to induce perturbation of vesicle membranes [57], [61]–[64]. We recently reported that the endosomolytic HA2 tag increases cellular uptake, accelerates endosomal escape, and promotes the even cytosolic distribution of endocytosed CPP-containing RFPs in human A549 cells [65]. The working concentrations of the cumbersome bacteria-produced R9-HA2-mCherry [65] and synthetic IR9-FITC were 30 and 5 µM, respectively. The relative transduction efficiency of 5 µM of IR9-FITC was much higher than that of 5 µM of R9-HA2-mCherry, whereas similar transduction efficiencies were noticed at high concentrations (both 30 and 60 µM) of R9-HA2-mCherry and IR9-FITC. Less cytotoxicity was detected with 30 µM of R9-HA2-mCherry; however, the same concentration of IR9 reduced cell viability.

Membrane potential plays a critical role in the internalization of arginine-rich CPPs into cells [66], [67]. We hypothesized that the charge state of IR9/cargo complexes can influence the efficiency of CPP-mediated cellular internalization. Protein transduction of CPP-mediated cargo delivery can be envisioned as a three-step process: first, binding to cellular membranes; second, penetration into cells; and third, release into cytoplasm or specific organelles [6], [58]. The first step of cellular uptake is initiated by electrostatic interactions between CPP/cargo complexes and negatively charged plasma membranes [58]. For instance, surface charge is a major determinant of how gold nanoparticles impact cellular processes, such as cell morphology, mitochondrial function, intracellular calcium levels and cytotoxicity [68]. Both positively and negatively charged gold particles are cytotoxic, with the negative ones being more toxic. Recently, electropositive zeta-potentials of nanodiamond particles were found to vary greatly depending on nanoparticle size, methods of production and treatment, surface structure and other properties [39], [69]. In our gel retardation assay, IR9/QD complexes attained a plateau of complex formation at a molecular ratio of 60 (Fig. 2), although higher transduction efficiencies were attained with higher molecular ratios, such as 120 (Fig. 3D and 4A). The transduction efficiency of IR9/cargo complexes correlated well with the magnitude of electropositive zeta-potentials of IR9/cargo complexes (Fig. 7). Electropositivity at or above 25 mV can be taken as an arbitrary value separating low-charged surfaces from highly-charged surfaces that contributes to suspension stability in colloidal systems [70]. Hence, this electrostatic property that governs CPP/cargo complex interactions with the negatively charged plasma membranes appears to be the key factor in determining transduction efficiency, rather than simple complex formation per se (as indicated by gel shift assay).

Though the understanding of cellular internalization of CPP/cargo complexes is still incompletely understood, the general belief is that most CPPs utilize two or multiple pathways for cellular entry [3]–[8]. Two major routes for cellular uptake of CPPs are the endocytic and nonendocytic pathways. Classical endocytosis is an energy-dependent pathway and includes both phagocytosis and pinocytosis [71], [72]. The nonendocytic route (also called direct membrane translocation, direct penetration, or pore-opening mechanism) is a rapid and energy-independent pathway [3], [73]. Antennapedia, R9 and Tat have been reported to simultaneously use at least three endocytic pathways: macropinocytosis, clathrin-mediated endocytosis and caveolae/lipid-raft-mediated endocytosis [74]. In this study, endocytosis was found to be one of the main routes for cellular uptake of IR9 and IR9/cargo complexes (Fig. 8).

The pathways involved in the internalization of QDs depend on their conjugated peptides or carriers [75]–[77]. CPP properties, CPP concentration, cargo characteristics, CPP/cargo complexing method, duration of transduction, serum concentration and composition of cell membrane have all been reported to influence cellular uptake efficiency and pathways of CPPs [56], [74], [78]–[81].

### Conclusions

A novel CPP, IR9, noncovalently interacts with QDs or DNAs to form stable complexes that are able to deliver into human A549 cells. Electrostatic interactions of IR9/cargo complexes with cellular membranes play a key role in cellular internalization. IR9 and IR9/cargo complexes may enter cells by endocytosis. IR9 includes the INF7 fusogenic domain which promotes the release of IR9/cargo complexes from endosomes. IR9 is relatively nontoxic and may be an excellent carrier of therapeutic cargoes in biomedical applications.

## References

[1] Rucevic M, Hixson D, Josic D (2011) Mammalian plasma membrane proteins as potential biomarkers and drug targets. Electrophoresis 32(13): 1549–64. 21706493 10.1002/elps.201100212

[2] Odds FC, Brown AJ, Gow NA (2003) Antifungal agents: mechanisms of action. Trends Microbiol 11(6): 272–9. 12823944 10.1016/s0966-842x(03)00117-3

[3] Madani F, Lindberg S, Langel U, Futaki S, Graslund A (2011) Mechanisms of cellular uptake of cell-penetrating peptides. J Biophys 2011: 414729. 21687343 10.1155/2011/414729 PMC3103903

[4] Mager I, Langel K, Lehto T, Eiriksdottir E, Langel U (2012) The role of endocytosis on the uptake kinetics of luciferin-conjugated cell-penetrating peptides. Biochim Biophys Acta 1818(3): 502–11. 22155257 10.1016/j.bbamem.2011.11.020

[5] Floren A, Mager I, Langel U (2011) Uptake kinetics of cell-penetrating peptides. Methods Mol Biol 683(2): 117–28. 21053126 10.1007/978-1-60761-919-2_9

[6] van den Berg A, Dowdy SF (2011) Protein transduction domain delivery of therapeutic macromolecules. Curr Opin Biotechnol 22(6): 888–93. 21489777 10.1016/j.copbio.2011.03.008

[7] Nakase I, Kobayashi S, Futaki S (2010) Endosome-disruptive peptides for improving cytosolic delivery of bioactive macromolecules. Biopolymers 94(6): 763–70. 20564044 10.1002/bip.21487

[8] Schmidt N, Mishra A, Lai GH, Wong GC (2010) Arginine-rich cell-penetrating peptides. FEBS Lett 584(9): 1806–13. 19925791 10.1016/j.febslet.2009.11.046

[9] Green M, Loewenstein PM (1988) Autonomous functional domains of chemically synthesized human immunodeficiency virus Tat trans-activator protein. Cell 55(6): 1179–88. 2849509 10.1016/0092-8674(88)90262-0

[10] Frankel AD, Pabo CO (1988) Cellular uptake of the Tat protein from human immunodeficiency virus. Cell 55(6): 1189–93. 2849510 10.1016/0092-8674(88)90263-2

[11] Vives E, Brodin P, Lebleu B (1997) A truncated HIV-1 Tat protein basic domain rapidly translocates through the plasma membrane and accumulates in the nucleus. J Biol Chem 272(25): 16010–7. 9188504 10.1074/jbc.272.25.16010

[12] Lindgren M, Langel U (2011) Classes and prediction of cell-penetrating peptides. Methods Mol Biol 683(1): 3–19. 21053118 10.1007/978-1-60761-919-2_1

[13] Gautam A, Singh H, Tyagi A, Chaudhary K, Kumar R, et al (2012) CPPsite: a curated database of cell penetrating peptides. Database 2012: bas015. 22403286 10.1093/database/bas015 PMC3296953

[14] Wagstff KM, Jans DA (2006) Protein transduction: cell penetrating peptides and their therapeutic applications. Curr Med Chem 13(12): 1371–87. 16719783 10.2174/092986706776872871

[15] Nakase I, Konishi Y, Ueda M, Saji H, Futaki S (2012) Accumulation of arginine-rich cell-penetrating peptides in tumors and the potential for anticancer drug delivery *in vivo* . J Control Release 159(2): 181–8. 22285548 10.1016/j.jconrel.2012.01.016

[16] Gump JM, Dowdy SF (2007) Tat transduction: the molecular mechanism and therapeutic prospects. Trends Mol Med 13(10): 443–8. 17913584 10.1016/j.molmed.2007.08.002

[17] Wadia JS, Dowdy SF (2002) Protein transduction technology. Curr Opin Biotechnol 13(1): 52–6. 11849958 10.1016/s0958-1669(02)00284-7

[18] Chang M, Chou JC, Lee HJ (2005) Cellular internalization of fluorescent proteins via arginine-rich intracellular delivery peptide in plant cells. Plant Cell Physiol 46(3): 482–8. 15695452 10.1093/pcp/pci046

[19] Wang YH, Chen CP, Chan MH, Chang M, Hou YW, et al (2006) Arginine-rich intracellular delivery peptides noncovalently transport protein into living cells. Biochem Biophys Res Commun 346(3): 758–67. 16781666 10.1016/j.bbrc.2006.05.205

[20] Chang M, Chou JC, Chen CP, Liu BR, Lee HJ (2007) Noncovalent protein transduction in plant cells by macropinocytosis. New Phytol 174(1): 46–56. 17335496 10.1111/j.1469-8137.2007.01977.x

[21] Liu K, Lee HJ, Leong SS, Liu CL, Chou JC (2007) A bacterial indole-3-acetyl-L-aspartic acid hydrolase inhibits mung bean (*Vigna radiata* L.) seed germination through arginine-rich intracellular delivery. J Plant Growth Regul 26(3): 278–84.

[22] Hou YW, Chan MH, Hsu HR, Liu BR, Chen CP, et al (2007) Transdermal delivery of proteins mediated by non-covalently associated arginine-rich intracellular delivery peptides. Exp Dermatol 16(12): 999–1006. 18031459 10.1111/j.1600-0625.2007.00622.x

[23] Liu BR, Chou JC, Lee HJ (2008) Cell membrane diversity in noncovalent protein transduction. J Membr Biol 222(1): 1–15. 18288433 10.1007/s00232-008-9096-6

[24] Hu JW, Liu BR, Wu CY, Lu SW, Lee HJ (2009) Protein transport in human cells mediated by covalently and noncovalently conjugated arginine-rich intracellular delivery peptides. Peptides 30(9): 1669–78. 19524630 10.1016/j.peptides.2009.06.006

[25] Lu SW, Hu JW, Liu BR, Lee CY, Li JF, et al (2010) Arginine-rich intracellular delivery peptides synchronously deliver covalently and noncovalently linked proteins into plant cells. J Agric Food Chem 58(4): 2288–94. 20092251 10.1021/jf903039j

[26] Liu BR, Huang YW, Lee HJ (2013) Mechanistic studies of intracellular delivery of proteins by cell-penetrating peptides in cyanobacteria. BMC Microbiol 13: 57. 23497160 10.1186/1471-2180-13-57 PMC3637573

[27] Chen CP, Chou JC, Liu BR, Chang M, Lee HJ (2007) Transfection and expression of plasmid DNA in plant cells by an arginine-rich intracellular delivery peptide without protoplast preparation. FEBS Lett 581(9): 1891–7. 17433309 10.1016/j.febslet.2007.03.076

[28] Li JF, Huang Y, Chen RL, Lee HJ (2010) Induction of apoptosis by gene transfer of human TRAIL mediated by arginine-rich intracellular delivery peptides. Anticancer Res 30(6): 2193–202. 20651369

[29] Lee CY, Li JF, Liou JS, Charng YC, Huang YW, et al (2011) A gene delivery system for human cells mediated by both a cell-penetrating peptide and a *piggyBac* transposase. Biomaterials 32(26): 6264–76. 21636125 10.1016/j.biomaterials.2011.05.012

[30] Dai YH, Liu BR, Chiang HJ, Lee HJ (2011) Gene transport and expression by arginine-rich cell-penetrating peptides in *Paramecium* . Gene 489(2): 89–97. 21925248 10.1016/j.gene.2011.08.011

[31] Chen YJ, Liu BR, Dai YH, Lee CY, Chan MH, et al (2012) A gene delivery system for insect cells mediated by arginine-rich cell-penetrating peptides. Gene 493(2): 201–210. 22173105 10.1016/j.gene.2011.11.060

[32] Liu BR, Lin MD, Chiang HJ, Lee HJ (2012) Arginine-rich cell-penetrating peptides deliver gene into living human cells. Gene 505(1): 37–45. 22669044 10.1016/j.gene.2012.05.053

[33] Liu MJ, Chou JC, Lee HJ (2013) A gene delivery method mediated by three arginine-rich cell-penetrating peptides in plant cells. Adv Stud Biol 5(2): 71–88.

[34] Wang YH, Hou YW, Lee HJ (2007) An intracellular delivery method for siRNA by an arginine-rich peptide. J Biochem Biophys Methods 70(4): 579–86. 17320189 10.1016/j.jbbm.2007.01.010

[35] Liu BR, Li JF, Lu SW, Lee HJ, Huang YW, et al (2010) Cellular internalization of quantum dots noncovalently conjugated with arginine-rich cell-penetrating peptides. J Nanosci Nanotech 10(10): 6534–43. 10.1166/jnn.2010.2637 PMC2999506 21137758

[36] Liu BR, Huang YW, Chiang HJ, Lee HJ (2010) Cell-penetrating peptide-functionized quantum dots for intracellular delivery. J Nanosci Nanotechnol 10(12): 7897–905. 21121277 10.1166/jnn.2010.3012 PMC2999507

[37] Xu Y, Liu BR, Lee HJ, Shannon KS, Winiarz JG, et al (2010) Nona-arginine facilitates delivery of quantum dots into cells via multiple pathways. J Biomed Biotechnol 2010: 948543. 21048930 10.1155/2010/948543 PMC2965432

[38] Liu BR, Huang YW, Winiarz JG, Chiang HJ, Lee HJ (2011) Intracellular delivery of quantum dots mediated by a histidine- and arginine-rich HR9 cell-penetrating peptide through the direct membrane translocation mechanism. Biomaterials 32(13): 3520–37. 21329975 10.1016/j.biomaterials.2011.01.041

[39] Liu BR, Chiang HJ, Huang YW, Chan MH, Chen HH, et al (2013) Cellular internalization of quantum dots mediated by cell-penetrating peptides. Pharm Nanotechnol 1(2): 151–61.

[40] Ziegler A, Nervi P, Durrenberger M, Seelig J (2005) The cationic cell-penetrating peptide CPP (Tat) derived from the HIV-1 protein Tat is rapidly transported into living fibroblasts: optical, biophysical, and metabolic evidence. Biochemistry 44(1): 138–48. 15628854 10.1021/bi0491604

[41] Tunnemann G, Ter-Avetisyan G, Martin RM, Stockl M, Herrmann A, et al (2008) Live-cell analysis of cell penetration ability and toxicity of oligo-arginines. J Pept Sci 14(4): 469–76. 18069724 10.1002/psc.968

[42] Kilk K, Mahlapuu R, Soomets U, Langel U (2009) Analysis of *in vitro* toxicity of five cell-penetrating peptides by metabolic profiling. Toxicology 265(3): 87–95. 19799958 10.1016/j.tox.2009.09.016

[43] Suhorustsenko J, Oskolkov N, Arukuusk P, Kurrikoff K, Eriste E, et al (2011) Cell-penetrating peptides, PepFects, show no evidence of toxicity and immunogenicity *in vitro* and *in vivo* . Bioconjug Chem 22(11): 2255–62. 21978260 10.1021/bc200293d

[44] Ekimov AI, Onushchenko AA (1981) Quantum size effect in three-dimensional microscopic semiconductor crystals. J Exp Theor Phys Lett 34(6): 345–9.

[45] Mattoussi H, Palui G, Na HB (2012) Luminescent quantum dots as platforms for probing *in vitro* and *in vivo* biological processes. Adv Drug Deliv Rev 64(2): 138–66. 21982955 10.1016/j.addr.2011.09.011

[46] Chen F, Gerion D (2004) Fluorescent CdSe/ZnS nanocrystal-peptide conjugates for long-term, nontoxic imaging and nuclear targeting in living cells. Nano Lett 4(10): 1827–32.

[47] Michalet X, Pinaud FF, Bentolila LA, Tsay JM, Doose S, et al (2005) Quantum dots for live cells, *in vivo* imaging, and diagnostics. Science 307(5709): 538–44. 15681376 10.1126/science.1104274 PMC1201471

[48] Shao L, Gao Y, Yan F (2011) Semiconductor quantum dots for biomedical applications. Sensors 11(12): 11736–51. 22247690 10.3390/s111211736 PMC3252007

[49] Delehanty JB, Mattoussi H, Medintz IL (2009) Delivering quantum dots into cells: strategies, progress and remaining issues. Anal Bioanal Chem 393(4): 1091–105. 18836855 10.1007/s00216-008-2410-4

[50] Xue FL, Chen JY, Guo J, Wang CC, Yang WL, et al (2007) Enhancement of intracellular delivery of CdTe quantum dots (QDs) to living cells by Tat conjugation. J Fluoresc 17(2): 149–54. 17203403 10.1007/s10895-006-0152-2

[51] Koshman YE, Waters SB, Walker LA, Los T, de Tombe P, et al (2008) Delivery and visualization of proteins conjugated to quantum dots in cardiac myocytes. J Mol Cell Cardiol 45(6): 853–6. 18835396 10.1016/j.yjmcc.2008.08.006 PMC2639397

[52] Wei Y, Jana NR, Tan SJ, Ying JY (2009) Surface coating directed cellular delivery of Tat-functionalized quantum dots. Bioconjug Chem 20(9): 1752–8. 19681598 10.1021/bc8003777

[53] Takeuchi T, Kosuge M, Tadokoro A, Sugiura Y, Nishi M, et al (2006) Direct and rapid cytosolic delivery using cell-penetrating peptides mediated by pyrenebutyrate. ACS Chem Biol 1(5): 299–303. 17163758 10.1021/cb600127m

[54] Gurtovenko AA, Anwar J (2007) Modulating the structure and properties of cell membranes: the molecular mechanism of action of dimethyl sulfoxide. J Phys Chem B 111(35): 10453–60. 17661513 10.1021/jp073113e

[55] Yang S, Coles DJ, Esposito A, Mitchell DJ, Toth I, et al (2009) Cellular uptake of self-assembled cationic peptide-DNA complexes: multifunctional role of the enhancer chloroquine. J Control Release 135(2): 159–65. 19171168 10.1016/j.jconrel.2008.12.015

[56] Wada JS, Stan RV, Dowdy SF (2004) Transducible Tat-HA fusogenic peptide enhances escape of TAT-fusion proteins after lipid raft macropinocytosis. Nat Med 10(3): 310–5. 14770178 10.1038/nm996

[57] El-Sayed A, Futaki S, Harashima H (2009) Delivery of macromolecules using arginine-rich cell-penetrating peptides: ways to overcome endosomal entrapment. AAPS J 11(1): 13–22. 19125334 10.1208/s12248-008-9071-2 PMC2664872

[58] Noguchi H, Matsushita M, Kobayashi N, Levy MF, Matsumoto S (2010) Recent advances in protein transduction technology. Cell Transplant 19(6): 649–54. 20525440 10.3727/096368910X508744

[59] Plank C, Oberhauser B, Mechtler K, Koch C, Wagner E (1994) The influence of endosome-disruptive peptides on gene transfer using synthetic virus-like gene transfer systems. J Biol Chem 269(17): 12918–24. 8175709

[60] Siennicka J, Gut W, Zuk A, Litwinska B (2003) Cytotoxicity of DMSO for MRC5, Chang liver and CV1 cells evaluated *in vitro* by LK, MTT and NR assays. Med Dosw Mikrobiol 55(2): 157–64. 14577195

[61] Michiue H, Tomizawa K, Wei FY, Matsushita M, Lu YF, et al (2005) The NH2 terminus of influenza virus hemagglutinin-2 subunit peptides enhances the antitumor potency of polyarginine-mediated p53 protein transduction. J Biol Chem 280(9): 8285–9. 15611109 10.1074/jbc.M412430200

[62] Sugita T, Yoshikawa T, Mukai Y, Yamanada N, Imai S, et al (2007) Improved cytosolic translocation and tumor-killing activity of Tat-shepherdin conjugates mediated by co-treatment with Tat-fused endosome-disruptive HA2 peptide. Biochem Biophys Res Commun 363(4): 1027–32. 17923117 10.1016/j.bbrc.2007.09.077

[63] El-Sayed A, Masuda T, Khalil I, Akita H, Harashima H (2009) Enhanced gene expression by a novel stearylated INF7 peptide derivative through fusion independent endosomal escape. J Control Release 138(2): 160–7. 19465073 10.1016/j.jconrel.2009.05.018

[64] Ye SF, Tian MM, Wang TX, Ren L, Wang D, et al (2012) Synergistic effects of cell-penetrating peptide Tat and fusogenic peptide HA2-enhanced cellular internalization and gene transduction of organosilica nanoparticles. Nanomedicine 8(6): 833–41. 22033082 10.1016/j.nano.2011.10.003

[65] Liou JS, Liu BR, Martin AL, Huang YW, Chiang HJ, et al (2012) Protein transduction in human cells is enhanced by cell-penetrating peptides fused with an endosomolytic HA2 sequence. Peptides 37(2): 273–84. 22898256 10.1016/j.peptides.2012.07.019 PMC9616647

[66] Wender PA, Galliher WC, Goun EA, Jones LR, Pillow TH (2008) The design of guanidinium-rich transporters and their internalization mechanisms. Adv Drug Deliv Rev 60(4–5): 452–72. 18164781 10.1016/j.addr.2007.10.016 PMC2533582

[67] Hirose H, Takeuchi T, Osakada H, Pujals S, Katayama S, et al (2012) Transient focal membrane deformation induced by arginine-rich peptides leads to their direct penetration into cells. Mol Ther 20(5): 984–93. 22334015 10.1038/mt.2011.313 PMC3345973

[68] Schaeublin NM, Braydich-Stolle LK, Schrand AM, Miller JM, Hutchison J, et al (2011) Surface charge of gold nanoparticles mediates mechanism of toxicity. Nanoscale 3(2): 410–20. 21229159 10.1039/c0nr00478b

[69] Schrand AM, Hens ASC, Shenderova OA (2009) Nanodiamon particles: properties and perspectives for bioapplications. Criti Rev Solid State Mater Sci 34(1–2): 18–74.

[70] Hanaor D, Michelazzi M, Leonelli C, Sorrell CC (2012) The effects of carboxylic acids on the aqueous dispersion and electrophorestic deposition of ZrO_2_ . J Eur Ceram Soc 32(1): 235–44.

[71] Conner SD, Schmid SL (2003) Regulated portals of entry into the cell. Nature 422(6927): 37–44. 12621426 10.1038/nature01451

[72] Iversen TG, Skotland T, Sandvig K (2011) Endocytosis and intracellular transport of nanoparticles: present knowledge and need for future studies. Nano Today 6(2): 176–85.

[73] Jiao CY, Delaroche D, Burlina F, Alves ID, Chassaing G, et al (2009) Translocation and endocytosis for cell-penetrating peptides internalization. J Biol Chem 284(49): 33957–65. 19833724 10.1074/jbc.M109.056309 PMC2797166

[74] Duchardt F, Fotin-Mleczek M, Schwarz H, Fischer R, Brock R (2007) A comprehensive model for the cellular uptake of cationic cell-penetrating peptides. Traffic 8(7): 848–66. 17587406 10.1111/j.1600-0854.2007.00572.x

[75] Bharali DJ, Lucey DW, Jayakumar H, Pudavar HE, Prasad PN (2005) Folate-receptor-mediated delivery of InP quantum dots for bioimaging using confocal and two-photon microscopy. J Am Chem Soc 127(32): 11364–71. 16089466 10.1021/ja051455x

[76] Tunnemann G, Martin RM, Haupt S, Patsch C, Edenhofer F, et al (2006) Cargo-dependent mode of uptake and bioavailability of Tat-containing proteins and peptides in living cells. FASEB J 20(11): 1775–84. 16940149 10.1096/fj.05-5523com

[77] Ruan G, Agrawal A, Marcus AI, Nie S (2007) Imaging and tracking of Tat peptide-conjugated quantum dots in living cells: new insights into nanoparticle uptake, intracellular transport, and vesicle shedding. J Am Chem Soc 129(47): 14759–66. 17983227 10.1021/ja074936k

[78] Maiolo JR, Ferrer M, Ottinger EA (2005) Effects of cargo molecules on the cellular uptake of arginine-rich cell-penetrating peptides. Biochim biophys Acta 1712(2): 161–72. 15935328 10.1016/j.bbamem.2005.04.010

[79] Kosuge M, Takeuchi T, Nakase I, Jones AT, Futaki S (2008) Cellular internalization and distribution of arginine-rich peptides as a function of extracellular peptide concentration, serum, and plasma membrane associated proteoglycans. Bioconjug Chem 19(3): 656–64. 18269225 10.1021/bc700289w

[80] Bolhassani A (2011) Potential efficacy of cell-penetrating peptides for nucleic acid and drug delivery in cancer. Biochim. Biophys. Acta 1816(2): 232–46. 10.1016/j.bbcan.2011.07.006 21840374

[81] Liu BR, Huang YW, Chiang HJ, Lee HJ (2013) Primary effectors in the mechanisms of transmembrane delivery of arginine-rich cell-penetrating peptides. Adv Stud Biol 5(1): 11–25.

